# Editorial: The role of ubiquitination in disease development, progression, and prognosis

**DOI:** 10.3389/fimmu.2025.1595755

**Published:** 2025-04-24

**Authors:** Mingyang Xue, Wenyi Jin

**Affiliations:** ^1^ School of Medicine, Kunming University of Science and Technology, Kunming, China; ^2^ Department of Biomedical Sciences, City University of Hong Kong, Hong Kong, Hong Kong SAR, China; ^3^ Department of Orthopaedics, Renmin Hospital of Wuhan University, Wuhan University, Wuhan, China

**Keywords:** ubiquitination, tumor cells, immune regulation, disease development, disease progression, tumor microenvironment

Ubiquitination, the covalent attachment of ubiquitin to target proteins, has emerged as a pivotal mechanism governing numerous cellular processes implicated in disease onset and progression. This Research Topic set out to explore how altered ubiquitination shapes pathogenesis, influences immune and inflammatory pathways, and offers potential avenues for novel therapeutic strategies. It has brought together articles that examine these roles from multiple angles, spanning gastrointestinal malignancies, immune-related conditions, metabolic disorders, and beyond. Here, we highlight the contributions of these published works and place them into the broader context of ubiquitin biology and its clinical implications.

One of the article to this Research Topic, Huang et al. dissects the complexity of gastrointestinal tumors by exploring how mitochondria, crucial for cellular energy supply and apoptosis regulation, are tightly controlled by ubiquitin-dependent processes. Mitochondrial biogenesis, mitophagy, and fission-fusion dynamics all rely on substrate proteins whose modification by E3 ubiquitin ligases determines their stability and location. When aberrant ubiquitination perturbs these processes, tumor cells gain proliferative advantages and become more resistant to apoptosis. By illuminating how specific E3 ligases and deubiquitinating enzymes (DUBs) target mitochondrial regulatory factors, the authors shed light on new strategies that might restore correct ubiquitin-mediated signaling and improve responses to therapies in gastrointestinal cancers.

Another study in this Research Topic, Deng et al. highlight the fine-tuned mechanisms by which ubiquitination modulates the cGAS-STING pathway, a critical cytosolic DNA-sensing axis in innate immunity. This research demonstrates how over- or under-ubiquitination of cGAS, STING, or their auxiliary factors leads to either exaggerated or dampened immune activation. The authors emphasize that in cancer and autoimmune states, rebalancing cGAS-STING signaling through targeted manipulation of ubiquitin ligases or DUBs holds promise. Their work contributes to a deeper understanding of how immune homeostasis might be re-established in disorders marked by a failure to either properly mount or properly resolve inflammation.

The interlink between neuroinflammation, sensory processing, and proteostasis also appears in this Research Topic, as Zhu et al. delve into the pathobiology of migraine. By investigating relevant ubiquitinating enzymes that govern pro-inflammatory mediators and nociceptive signals, the authors reveal that the ubiquitin-proteasome system can alter the expression and turnover of calcitonin gene-related peptide and MAPK/NF-κB components. Their findings advance our grasp of the molecular underpinnings of migraine chronicity and point to promising molecular targets for treatments aimed at pain prevention rather than mere symptomatic relief.

Sepsis, a hyper-inflammatory state triggered by pathogens and sustained by damage-associated molecular patterns, is further addressed through an examination of ubiquitin’s role in regulating pathways leading to cytokine storm and cell death. Li et al. illustrate how ubiquitin-dependent modifications of RIPK1 and NLRP3, central players in necroptosis and pyroptosis, allow the immune system to either escalate or temper the inflammatory reaction. They also underscore how dysregulation of deubiquitinating enzymes can tip the delicate balance, culminating in excessive or persistent inflammation that damages multiple organs. Such insights suggest that selective E3 ligase inhibitors or DUB modulators could become part of emerging therapies to minimize sepsis-related morbidity.

Contributors like Cheng et al. to this Research Topic also examine how metabolic disorders, including metabolic dysfunction-associated steatohepatitis and chronic viral hepatitis, converge on common ubiquitin-related pathways. Differential gene expression analyses in these conditions pinpoint an overrepresentation of immune and inflammatory routes, hinting that aberrant ubiquitination influences the fate of key proteins like STAT1 or CCL2. Connecting these genes to changes in protein stability not only enhances our understanding of disease progression but also opens the door to potential ubiquitin-targeted interventions. An example is the demonstration that clinically approved agents can suppress aspects of the pathogenic signature by recalibrating protein turnover, thereby offering encouraging leads for more personalized treatment strategies.

A further set of findings focuses on hepatocellular carcinoma and details how subsets of E3 ubiquitin ligases orchestrate the turnover of oncogenes, tumor suppressors, and immune-regulatory factors. The authors Wang et al. describe how these ligases, belonging to the RING, HECT, or RBR families, can directly shape tumor proliferation, apoptosis, and metastatic potential. By fine-tuning checkpoints and immune cell activation, ubiquitination also profoundly impacts how the tumor microenvironment responds to immunotherapy. These insights strengthen the rationale that targeting E3 ligases in combination with checkpoint inhibitors or other modalities could enhance treatment efficacy and perhaps overcome therapeutic resistance.

The integrative effect of ubiquitination on immune function and cellular homeostasis is showcased further in work that explores rheumatoid arthritis (RA). There, researchers Fu et al. highlight how abnormal tagging of proteins in immune and synovial cells can amplify inflammatory cascades and tissue destruction. In particular, the interplay between metabolic changes, for example lactylation, and ubiquitin-dependent degradation of cell-cycle regulators underscores that RA pathogenesis is fueled by both immunological and metabolic shifts. By pinpointing E3 ligases, such as BIRC3, which drive fibroblast-like synoviocyte proliferation and inflammatory signaling, the authors Meng et al. argue that selective inhibition of these ligases might hold the key to halting joint damage and improving disease outcomes.

Concluding the array of contributions are data on mesenchymal stem cells and inflammatory bowel disease, emphasizing how interventions that recalibrate ubiquitination can restore gut immune homeostasis. The authors Liao et al. propose that by influencing the stability of critical signaling agents in the inflamed intestine, stem-cell-based therapies may achieve a more targeted and lasting control of chronic inflammation. These findings open further discussion about how precisely timed or localized manipulation of ubiquitin pathways could complement existing immunomodulatory treatments for inflammatory bowel disease.

Collectively, the articles in this Research Topic underscore how ubiquitination has evolved from a niche protein-tagging mechanism into a unifying framework that shapes myriad facets of disease biology. In addition to unveiling novel E3 ligases and DUBs that modulate pathogenesis, these studies advocate for therapeutic strategies that correct dysregulated ubiquitination. The growing roster of small molecule inhibitors and biologics aimed at specific steps in the ubiquitination cascade offers unprecedented opportunities to alter disease trajectories in cancer, autoimmune disorders, sepsis, neurological diseases, and beyond. As the field moves forward, a deeper characterization of ubiquitin modifications in human tissues should guide the discovery of more refined diagnostic and therapeutic tools, expanding the horizon of precision medicine.

